# Clustering and Beamforming for Efficient Communication in Wireless Sensor Networks

**DOI:** 10.3390/s16081334

**Published:** 2016-08-20

**Authors:** Francisco Porcel-Rodríguez, Juan Valenzuela-Valdés, Pablo Padilla, Francisco Luna-Valero, Rafael Luque-Baena, Miguel Ángel López-Gordo

**Affiliations:** 1 Department of Signal Theory, Telematics and Communications—CITIC, University of Granada, 18071 Granada, Spain; franciscoporcel@correo.ugr.es (F.P.-R.); pablopadilla@ugr.es (P.P.); malg@ugr.es (M.Á.L.-G.); 2 Department of Computer Science and Programming Languages, University of Malaga, 29071 Malaga, Spain; flv@lcc.uma.es; 3 Department of Computer and Telematics Systems Engineering, University of Extremadura, 06800 Merida, Spain; rmluque@unex.es

**Keywords:** wireless sensors networks, energy efficiency, beamforming, optimization techniques

## Abstract

Energy efficiency is a critical issue for wireless sensor networks (WSNs) as sensor nodes have limited power availability. In order to address this issue, this paper tries to maximize the power efficiency in WSNs by means of the evaluation of WSN node networks and their performance when both clustering and antenna beamforming techniques are applied. In this work, four different scenarios are defined, each one considering different numbers of sensors: 50, 20, 10, five, and two nodes per scenario, and each scenario is randomly generated thirty times in order to statistically validate the results. For each experiment, two different target directions for transmission are taken into consideration in the optimization process (*φ* = 0° and θ = 45°; *φ* = 45°, and θ = 45°). Each scenario is evaluated for two different types of antennas, an ideal isotropic antenna and a conventional dipole one. In this set of experiments two types of WSN are evaluated: in the first one, all of the sensors have the same amount of power for communications purposes; in the second one, each sensor has a different amount of power for its communications purposes. The analyzed cases in this document are focused on 2D surface and 3D space for the node location. To the authors’ knowledge, this is the first time that beamforming and clustering are simultaneously applied to increase the network lifetime in WSNs.

## 1. Introduction

**Figure 1:**
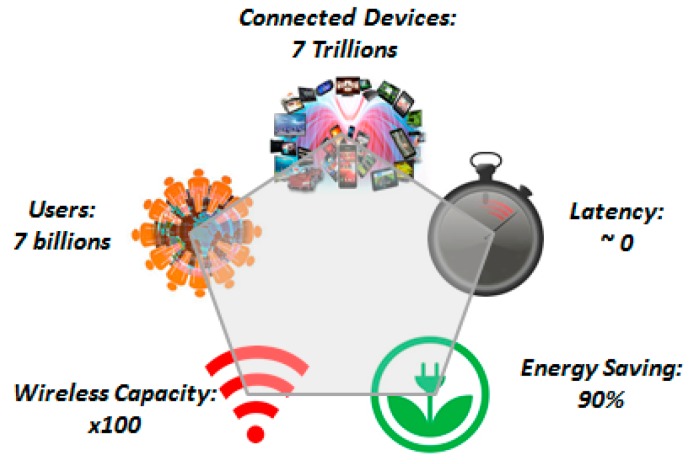
Design goals and requirements for 5G networks. (adapted from [3]).

The next generation 5G wireless communications are currently being developed [1,2]. Indeed, the first deployments of a 5G network are expected to be fully operating in 2020 [3]. The design goals of such systems are shown in Figure 1. These systems are conceived to provide very high data rates (typically Gbps), extremely low latency, manifold increase in base station capacity and significant improvement in users’ perceived quality of service (QoS), compared to current 4G LTE networks [4]. There are many innovative technologies that will be used to satisfy the demands of the massive volume of traffic and various devices: massive MIMO, orthogonal frequency-division multiple access (OFDMA), cloud radio access network (CRAN), software-defined networking (SDN), composite wireless infrastructures, flexible spectrum management, small cells, and heterogeneous network deployment, among others [5].

The research initiatives by industry and academia have identified eight major requirements [1] of the next generation 5G systems, with “the reduction in energy usage by almost 90%” being one of them. Green technologies are, thus, being considered in the standard bodies. As a consequence, energy efficiency is, therefore, a key issue for the design of these networks [6]. Efficiency, in general, and energy consumption, in particular, clearly involve some sort of optimization. The required technologies for the development of 5G systems, as well as the application scenarios, are quite numerous. One of the most potential use cases of 5G [4] is in wireless sensor networks (WSNs). The expected number of connected “things” (IoT: Internet of Things) will be 7 trillion [3]. Therefore, it is clear that energy preservation for WSNs is an issue of great concern with respect to network design and deployment, protocols, and configurations, trying to maximize the performance of the nodes.

One option is to reduce the energy consumption by optimizing the way the wireless communications take place. The problem has to cope with the air interface (the radio signals) and how the radiation pattern (beam) is set up to reach the desired QoS with minimal energy consumption. The technique that enables this energy-efficient wireless communication is beamforming [7], which consists of several coordinated antennas radiating jointly to generate a directive beam for covering a given area with very accurate precision. The goal is to reduce the energy consumption of the sensors by performing collaborative beamforming. This is a very important problem as the sensor nodes have limited power, and saving energy is critical. In this paper, the objective is to show that beamforming can be used in the context of WSNs to perform energy efficient communications, allowing the lifetime of this kind of distributed infrastructure to be steadily increased. To the best of our knowledge, this is the first approach in which the gain of the beamforming is accurately computed, in relation to the number of nodes, organized in different clusters in the WSN.

The paper is organized as follows: Section 2 describes the beamforming model used for a WSN; Section 3 shows the experimental set-up and pattern results; Section 4 provides the theoretical results, and in Section 5 the gain results and discussion of the results obtained are exposed; and the conclusions are outlined in Section 6.

## 2. Collaborative Beamforming in WSN

WSNs usually have the nodes arbitrarily located in a defined area. In many cases, this distribution is a random one. This has an influence in the data transmission when considering collaborative nodes; not being easy to maximize the global transmission capacity of the network. In this context, beamforming has arisen as a good strategy in WSNs for complete system optimization in terms of energy efficiency, and network capacity and reliability. The first approaches on this are dated only a few years ago. In [8], the authors prove the existence of an optimum feeding configuration for each individual element of the network when trying to maximize the transmission gain in a desired spatial direction. Moreover, in the results in [9] it is stated that 80% of the network energy is saved when applying beamforming. In [10], the authors provide a theoretical proof of the gain improvement if beamforming is applied. In this work, we compute iteratively the beamforming in an array of sensors to calculate the possible gain improvement based on this approach [11,12].

### 2.1. Beamforming

The beamforming techniques are based on the definition of a highly directive pattern in a desired direction, depending on the network operation conditions and the network transmitting necessities. Based on this, it is necessary to consider the field of each individual element contributing constructively in the desired pattern direction, and destructively in the rest of them, even provoking transmission nulls in some critical directions. The general pattern is made of the aggregation of the elementary ones, and five variables define the antenna array that the nodes form. These variables can be classified into two different categories, which are the radiating element nature and the spatial location:

1. Radiating element: the amplitude excitation of each unitary radiating element.
2. The phase excitation of each unitary radiating element.
3. The radiation pattern of each element, depending on the kind of antenna considered (dipole, isotropic ideal, etc.).

Spatial location:

1. The separation among elements of the array, in terms of wavelength distance.
2. The antenna array geometry, which may be linear, circular, rectangular, or spherical, among other possibilities.

Considering classical array theory, in terms of computation, the easiest and affordable manner of placing the elements is a 1D line or 2D surface in which the elements are uniformly separated and with the same excitation in both components: amplitude and phase. This simple configuration lets one obtain a variation in the radiation direction when acting progressively over the phase of each element. Unfortunately, this is not the case of WSN networks, whose nodes are distributed in space randomly. However, there is still enough room to achieve benefits from applying beamforming, although it becomes a harder task to find the optimal configuration.

### 2.2. WSNs Scenario Model

The experimental setup considered in this work implies a random node deployment in a squared area or cubic volume with dimensions of 30 on each side. The node location is based on a uniform distribution in both 2D coordinates or 3D coordinates, with no movement once the nodes are settled down. This approach is widely used in the definition of WSN experimental scenarios, as can be found in [7] or [8]. In this scenario, each node has limited power autonomy, which must fulfill the operation requirements for both data transmitting to the High Energy Communication Node (HECN) and environment sensing.

The nodes are also equipped with two different antenna types providing different antenna patterns. Although it is typically assumed that the total available power is the same at each sensor, the distribution of power devoted to sensing or to data transmission can be modeled to be diverse. In this work, it is assumed that the node consumption for each task is commanded by random uniformly distributed variable of value [0, 1]. Thus, the available energy at sensor *x* (*x* = 1, 2, …, max_sensors), *Ea_x_*, is the following:

(1) $$E_{a_{x}}=E_{t}\cdot F_{x}$$

where *E_t_* is the total available sensor energy and *F_x_* is the uniformly distributed variable described above. Additionally, the energy consumed by each sensor (*Ec_x_*) is:

(2) $$E_{C_{x}}=P_{tx\_x}\cdot t_{x}$$

where *P_tx_x_* is the amount of power devoted to transmit the data in sensor *x* and *t_x_* is the transmission time. Notice that the node consumption can be also expressed in terms of power. Thus, the available power, *Pa_x_*, is provided by:

(3) $$P_{a_{x}}=P_{tx\_x}\cdot F_{x}$$

As it can be noticed, the maximum lifetime, *t_life_x_*, for the sensors as a whole, is obtained when the energy consumption is equal to the available energy:

(4) $$E_{t}\cdot F_{x}=P_{tx\_x}\cdot t_{life\_x}$$

As a consequence, the lifetime of each sensor can be written as follow:

(5) $$t_{life\_x}=\frac{E_{t}\cdot F_{x}}{P_{tx\_x}}$$

At this point, it must be considered that *P_tx_x_* is the power needed to transmit data without considering beamforming. However, when beamforming is applied, *P_tx_x_*, which is related to the excitation in amplitude of each sensor, is multiplied by a gain effect provided by the beamforming. This gain effect depends on the final radiation pattern and may induce gain in some directions and loss in others. Thus, the power to transmit data to the receptor is:

(6) $$P_{tx\_x\_B\_A}=P_{tx\_x}\cdot GB\left(\theta ,\phi \right)$$

where *P*_*tx_*x_B_A_ is the excitation in amplitude of each sensor and *GB* is the global gain value at the different directions of transmission/reception. Thus, *P_tx_x_* considering beamforming is:

(7) $$P_{tx\_x}=\frac{P_{tx\_x\_B\_A}}{GB\left(\theta ,\phi \right)}$$

Finally, the lifetime when adding beamforming, *t*_*life*_*x*_B_, is:

(8) $$t_{life\_x\_B}=\frac{E_{t}\cdot F_{x}\cdot GB\left(\theta ,\phi \right)}{P_{tx\_x\_A\_B}}$$

The main interest in this work is focused on the maximization of the lifetime of the WSN as a whole, applying beamforming for the data transmission among the sensors. This is translated into a search of phase and amplitude configurations, *P_tx__*_x_B_, at each sensor that maximize the final outcome:

(9) $$\max\left\{t_{WSN}=min\left(t_{life\_x\_B}\right),\;x\epsilon \left[1,\text{max}\_sensors\right]\right\}$$

Notice that the sensor that first runs out of battery fixes the lifetime of the WSN.

### 2.3. Optimization Algorithm

In order to address the problem defined above in Equation (9), a genetic algorithm (GA) has been adopted as the optimization algorithm because they have shown good performance over a great variety of optimization problems [8]. GAs manage a pool of candidate solutions (the population), which represent tentative solutions of the target problem. In our case, these solutions are composed of the excitation (module and phase) of the sensors deployed in the WSN. That is, solutions are arrays (vectors) of real-coded values in which the modules of all sensors are arranged first, and their phases afterwards. Given such a solution, the lifetime of all of the sensors is computed with Equation (8) (using the corresponding modules and phases), and the minimum of such lifetimes is the objective to be maximized, as Equation (9) defines.

The GA population is iteratively improved by using genetic operators (selection, recombination, and mutation) that follows the idea of “survival of the fittest”, i.e., new solutions are generated in each generation and replace worse solutions in the population. The process of iterating through successive generations is called evolution, and ends when a termination condition is fulfilled.

The GA included in the optimization toolbox of Matlab^®^ (Natick, MA, USA) has been used. The default configuration of this GA and its standard settings are used: the population size has been set to 100; as genetic operators, remainder selection, heuristic crossover (with a probability of 0.8), and uniform mutation (probability of 0.1); finally, the stopping condition is to perform 100 generations. These parameter values are widely accepted and they are known to perform well over a variety scenarios [13].

## 3. Experimental Set-up and Pattern Results

The radiation patterns and their gains when applying beamforming are computed with [14], a Matlab toolbox^®^. Beamforming requires all of the nodes in the cluster to be synchronized, and this issue takes time: the higher the cluster size, the longer the sync time needs to be. In this work we have fixed five different scenarios regarding the number of sensors implied: 50, 20, 10, five, and two nodes per scenario. Additionally each scenario is randomly generated thirty times in order to statistically validate the results. For the scenarios of 50, 20, 10, five, and two nodes, a transmission time of 40% 30%, 20%, 15%, and 10% is used for synchronization, respectively. The time of transmission of the beamforming technique is multiplied by the number of sensors because every sensor needs to transmit the data of all sensors. In each experiment, two different transmission directions are being considered for the optimization process (*φ* = 0° and θ = 45°; *φ* = 45°, and θ = 45°). Then, the GA starts optimizing the antenna parameters installed in these sensors for performing the desired beamforming (a beam towards the HECN, which is located at the four different directions previously mentioned). Each scenario is evaluated for two different antennas, an ideal isotropic antenna and a conventional dipole one. In this set of experiments all the sensors are considered to be transmitting the same amount of power when acting in the network as a collaborative node.

### Pattern Results

**Figure 2:**
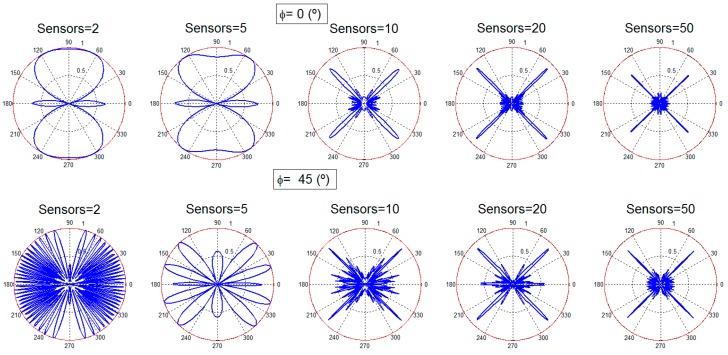
Radiation patterns for two, five, 10, 20 and 50 sensors, different search angles: isotropic antenna.

**Figure 3:**
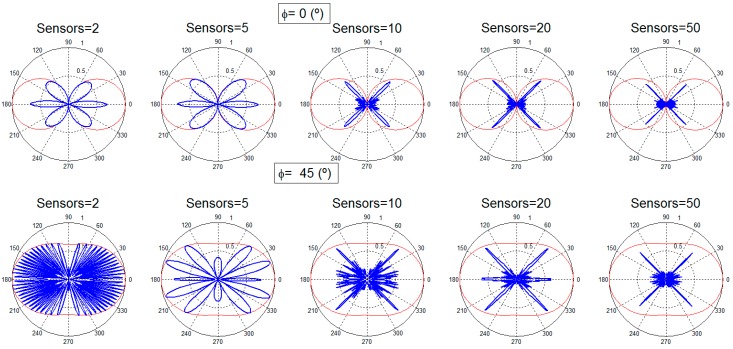
Radiation patterns for two, five, 10, 20 and 50 sensors, different search angles: dipole antenna.

Figure 2 and Figure 3 present the performance results. At each subfigure, it should be noticed that, although the desired transmission direction implies a particular θ and *φ* value, the subfigures provide the radiation pattern results for the particular *φ* and the entire θ range (360°). In this θ range, the desired θ direction can be easily identified.

Figure 2 provides the beamforming results for two, five, 10, 20, and 50 sensors with isotropic antennas for the two different angular configurations. It is observed that although the desired direction is always obtained, for a low number of sensors there is still a high amount of radiated power towards other directions. This limitation is logical regarding the reduced number of sensors considered. As the number of nodes is increased, the radiated power towards directions different from the desired one is reduced, as it is clearly identified in Figure 2. In the case of using a dipole (Figure 3), the dipole radiation pattern adds a limitation in the beam that points towards the desired direction. The final beam is, thus, affected by the radiation pattern of the dipole, reducing the global gain achieved, as expected. When the number of nodes is high (20 and 50 nodes), the desired beam becomes more directive, the number of the rest of the beams, and also their gain, is reduced.

## 4. Theoretical Gain Results

In order to analyze the gain results, four different scenarios have been proposed. In the first one (A) all of the sensors have the same power available for communications (this is the case of a WSN composed with the same sensor type) and the sensors are placed only in two dimensions. In the second scenario (B) the sensors have random available power for communications (this is the case of a WSN composed with different sensor types) and the sensors are again placed only in two dimensions. In the third scenario (C) all sensors have the same available power for the communications but the sensors are placed in three dimensions. In the fourth scenario (D) the sensors have random available power for communications and again the sensors are placed in three dimensions.

### 4.1. Theoretical Gain Results

**Figure 4:**
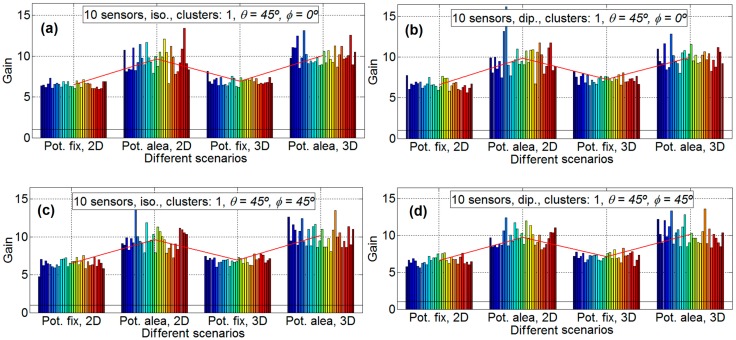
Gain results for 10 sensors. Isotropic ideal antenna, *φ* = 0° and θ = 45° in subfigure (**a**) (**upper left**), dipole antenna, *φ* = 0° and θ = 45° in subfigure (**b**) (**upper right**), isotropic ideal antenna, *φ* = 45° and θ = 45° in subfigure (**c**) (**lower left**) and dipole antenna, *φ* = 45° and θ = 45° in subfigure (**d**) (**lower right**).

**Figure 5:**
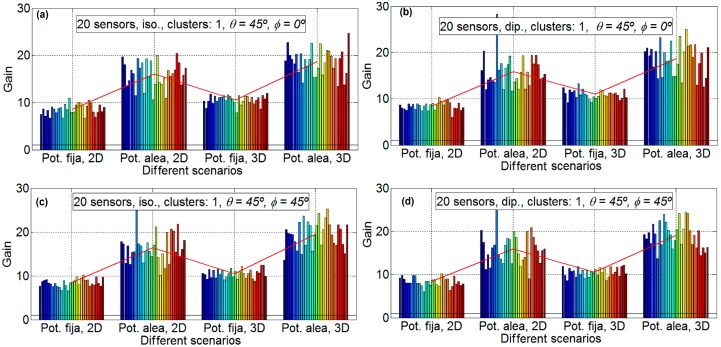
Gain results for 20 sensors. Isotropic ideal antenna, *φ* = 0° and θ = 45° in subfigure (**a**) (**upper left**), dipole antenna, *φ* = 0° and θ = 45° in subfigure (**b**) (**upper right**), isotropic ideal antenna, *φ* = 45° and θ = 45° in subfigure (**c**) (**lower left**) and dipole antenna, *φ* = 45° and θ = 45° in subfigure (**d**) (**lower right**).

**Figure 6:**
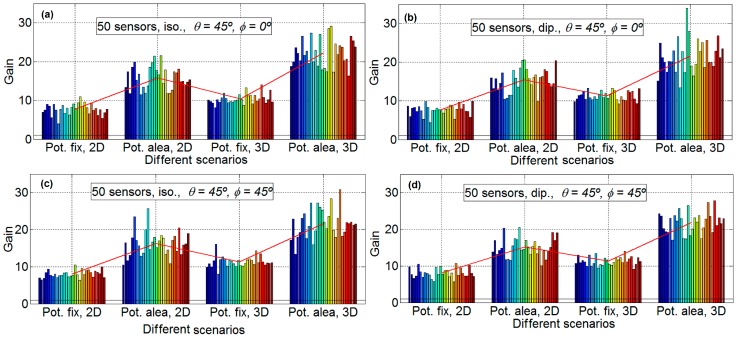
Gain results for 50 sensors. Isotropic ideal antenna, *φ* = 0° and θ = 45° in subfigure (**a**) (**upper left**), dipole antenna, *φ* = 0° and θ = 45° in subfigure (**b**) (**upper right**), isotropic ideal antenna, *φ* = 45° and θ = 45° in subfigure (**c**), (**lower left**) and dipole antenna, *φ* = 45° and θ = 45° in subfigure (**d**) (**lower right**).

In order to analyze the different gain factors implied in the energy efficiency in a WSN, in this subsection, the distance between the nodes and the HECN has not been taken into account. That is, the gain factor obtained is only due to the beamforming technique. The gain results are analyzed: Figure 4, Figure 5 and Figure 6 provide the gain values for the two different desired search angles, all scenarios and both antennas, with 10 sensors, 20 sensors, and 50 sensors, respectively. The gain values are computed in terms of the increment factor regarding the gain in the case beamforming is not used; that is, the gain obtained for a unitary node for the desired direction and provided by its antenna pattern.

Each experiment is repeated 30 times so that 30 different sets of sensor positions are defined, in order to statistically validate the results. In the result figures (Figure 4, Figure 5 and Figure 6) the subfigures have the following characteristics: isotropic ideal antenna (*φ* = 0° and θ = 45°) in Figure 4a (upper left), dipole antenna (*φ* = 0° and θ = 45°) in Figure 4b (upper right), isotropic ideal antenna (*φ* = 45° and θ = 45°) in subfigure c (lower left), and dipole antenna (*φ* = 45° and θ = 45°) in subfigure d (lower right). In all subfigures it is plotted with a red line the mean gain of each scenario. In Figure 4 it is shows the results for 10 nodes and 30 emulations for each scenario (four scenarios for each subfigure). The aspect of all subfigures is very similar; that is, the influence of the directions and the antenna is very limited. For example the mean gain for the scenario A, for Figure 4a is 6.50, for Figure 4b is 6.60, for Figure 4c is 6.51, and Figure 4d is 6.61, which means that the maximum difference between subfigures is only 0.11 (1.6%). However, the gain factor is different for the different scenarios, around 6.55 for scenario A; around 9.7 for scenario B; around 7 for scenario C; and around 10 for scenario D. This means that the scenarios with fixed power have less gain than the scenarios with sensors with different power. This is because the genetic algorithm tries to assign the node with lower power to the communication. The lower the power to conform the beamforming, the higher the lifetime of the WSN. For 10 nodes there exists little difference between 2D and 3D scenarios.

Figure 5 shows the results for 20 nodes and 30 emulations for each scenario (four scenarios for each subfigure). For 20 nodes, the aspect of all of the subfigures is also very similar, that is, the influence of the directions and the antenna is very limited. For example, the mean gain for scenario B, for Figure 5a is 16.03, for Figure 5b is 15.91, for Figure 5c is 16.41, and for Figure 5d is 15.98, which means that the maximum difference between subfigures is only 0.5 (3.0%). However, the gain factor is different for the different scenarios, around 8.4 for scenario A, around 16.2 for scenario B, around 10.7 for scenario C, and around 18.8 for scenario D. Again, the scenarios with fixed power have less gain than the scenarios with sensors with different power for the same reasons as that for 10 nodes. For 20 nodes there exists differences between the 2D and the 3D scenarios, and the gain factors for 2D scenarios are around 15% lower than the ones for 3D scenarios. This may be influenced by the major complexity of the calculations in the 3D case which may lead to non-optimal solutions when the GAs algorithms are applied.

Figure 6 show the results for 50 nodes and 30 emulations for each scenario (four scenarios for each subfigure). Again, for 50 nodes, the aspect of all the subfigures is very similar, which means that the influence of the directions and the antenna is very limited. For example, the mean gain for the scenario D, for Figure 6a is 22.25, for Figure 6b is 21.6, for Figure 6c is 21.66, and Figure 6d is 21.93, which implies that the maximum difference between subfigures is only the 0.65 (2.9%). As it is expected, the gain factor is again different for the different scenarios, around 7.75 for scenario A, around 15.8 for scenario B, around 10.3 for scenario C, and around 21.6 for scenario D. This means, in a similar way to the Figure 4 and Figure 5, that the scenarios with fixed power have less gain than the scenarios with sensors with different power. For 50 nodes there exist more differences between 2D and 3D scenarios, the gain factors for 2D scenarios are around a 30% less than for 3D scenarios for fixed power and around 40% less for sensors with random power for communications. It is clear from Figure 6 that the variability of scenarios with 50 sensors is high. For example, in subfigure b in scenario D, the emulation with the higher gain has a gain factor of 34 and the emulation with less gain has a gain factor of 13.4; that is, the emulation with less gain is only 39.4% than that of the emulation with more gain.

**Figure 7:**
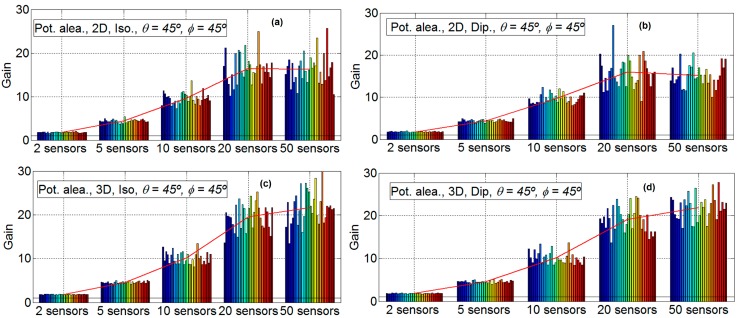
Gain results for two, five, 10, 20 and 50 sensors with *φ* = 45° and θ = 45°. Isotropic ideal antenna and scenario B in subfigure (**a**) (**upper left**), dipole antenna and scenario B in subfigure (**b**) (**upper right**) isotropic antenna and scenario D in subfigure (**c**) (**lower left**) and dipole antenna and scenario B in subfigure (**d**) (**lower right**).

**Figure 8:**
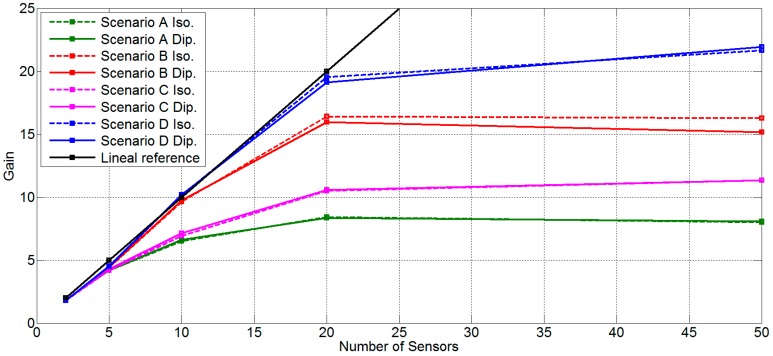
Results for search angle *φ* = 0°, θ = 45°, all scenarios and both antennas.

Figure 7 shows that, for the results considering two, five, 10, 20 and 50 nodes and 30 emulations for four scenarios (one scenario for each subfigure), the direction has little influence. Consequently, it is only shown for *φ* = 45° and θ = 45°. The subfigures have the following characteristics: isotropic ideal antenna and scenario B in Figure 7a (upper left), dipole antenna and scenario B in Figure 7b (upper right) and isotropic ideal antenna, scenario D in Figure 7c (lower left) and dipole antenna and scenario B in Figure 7d (lower right). Again, in all of the subfigures, the mean gain of each scenario is plotted with a red line. It is shown that the variability of the results increases with the number of sensors. With a low number of sensors (two and five) the standard deviation of results is low, and for a high number of sensors (20 and 50) the variability is very high. Additionally, in this figure, it is shown that the gain increase with the number of elements, except for 50 sensors. More details can be observed in Table 1 and Figure 8.

Table 1 shows the mean gain factor for all scenarios with two antenna and *φ* = 45° θ = 45°. Figure 8 shows the same data that is in Table 1 in a graphical mode. Moreover, a linear reference for visual performance it is added. In this figure it is possible to observe that, for a low number of sensors (two and five) all scenarios work well; the main gain factor is approximately equal to the number of sensors. For an intermediate number of sensors (10), only scenarios B and D obtain a mean gain factor near the number of sensors. Meanwhile, scenarios A and C lose more than 30% of the main gain factor with respect to Scenarios B and D. No great difference between 2D and 3D position is found. For 20 sensors, the unique scenario that has a mean gain factor similar to the number of sensors is scenario D. With 20 sensors there exist differences between all scenarios; 3D scenarios work better than 2D scenarios, and scenarios with sensors with random power have more possibilities to conform the beamforming than scenarios with sensors with fixed power. Finally, with 50 sensors, only 3D scenarios (C and D) increase the mean gain factor obtained with 20 sensors, and the increase is small and very far from the number of sensors.

The main reason that explains these results of the GA has to do with the size of the instances and the stopping condition of the algorithm, which has been kept the same in all the scenarios. As the number of sensors increase, the search space to be explored by the GA becomes much larger, but the sampling size (i.e., the number of function evaluations) is constant: 100 individuals × 100 generations = 10,000 evaluations. This clearly favors the small instances for which the GA is given more chance to find better quality solutions. Either increasing the population size of the GA, its number of generations to stop, or both would be required together with advanced diversity preservation mechanism that avoid the search to get stuck in local optima (premature convergence). In any case, there is room for improvement, especially when large instances are addressed.

From the point of view of the node synchronization, the synchronization of 50 sensors can imply a significant problem because all of the nodes must transmit at the same time. Additionally, the calculation of the amplitudes and phases is not a trivial aspect for a genetic algorithm because the number of variables to be optimized is equal to 100. Therefore, a good choice can be the clusterization of the WSN, when the number of sensors is high. This is discussed in the next section.

### 4.2. Cluster Gain Results

**Figure 9:**
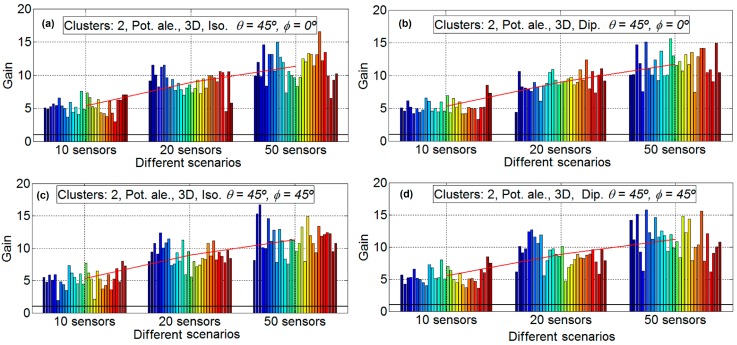
Gain results with two clusters, 10, 20 and 50 sensors, Scenario D. Isotropic antenna *φ* = 0° and θ = 45° in subfigure (**a**) (**upper left**), dipole antenna, *φ* = 0° and θ = 45° in subfigure (**b**) (**upper right**), isotropic antenna, *φ* = 45° and θ = 45° in subfigure (**c**) (**lower left**) and dipole antenna, *φ* = 45° and θ = 45° in subfigure (**d**) (**lower right**).

In this section, the sensor network has been divided into two clusters to study the behavior of the sensor network when the number of sensors is high. To split into two clusters of sensors, we have used the method of k-means, which is one of the simplest and popular, as explained in [15]: “One of the most popular and simple clustering algorithms, K-means, was first published in 1955. In spite of the fact that K-means was proposed over 50 years ago and thousands of clustering algorithms have been published since then, K-means is still widely used”. The procedure is as follows: first the K-means algorithm is applied to all sensors, obtaining two clusters, then, the beamforming technique is applied to the two clusters. To maintain the computational costs, a unique genetic algorithm has been applied in order to optimize the two clusters simultaneously, so that only one genetic algorithm optimizes the phases and amplitudes of the two clusters. Obviously, it would be a better strategy to apply two different genetic algorithms, one to each of the clusters. Figure 9 shows the results of performing optimization with two clusters, where 10, 20, and 50 sensors are simulated. All of the subfigures are for the Scenario D. The subfigures have the following characteristics: isotropic ideal antenna, *φ* = 0° and θ = 45° in subfigure a (upper left), dipole antenna, *φ* = 0° and θ = 45° in subfigure b (upper right), isotropic ideal antenna, *φ* = 45° and θ = 45° in subfigure c (lower left) and dipole antenna, *φ* = 45° and θ = 45° in subfigure D (lower right). Figure 9 shows that the gain factor is reduced (50%) when 10 and 20 sensors are simulated. This result is coherent since two clusters with half of the elements are being combined. As it has been stated in previous sections, the gain for scenario D is approximately equal to the number of elements in the range of 0–20. It is observed that the variability of the results is much higher when considering two clusters than when considering just one. However, with 50 sensors the gain achieved is about 12, not reaching the expected gain of 20. This gain value is expected because, with two clusters with more than 20 sensors, the gain at each cluster must be equal to the number of sensors in the cluster. The same explanation as in the previous section holds here. More sensors enlarge the search space to be explored by the GA, whilst is configuration remains the same concerning the number of function evaluations performed (sampling size).

## 5. Gain Results

**Figure 10:**
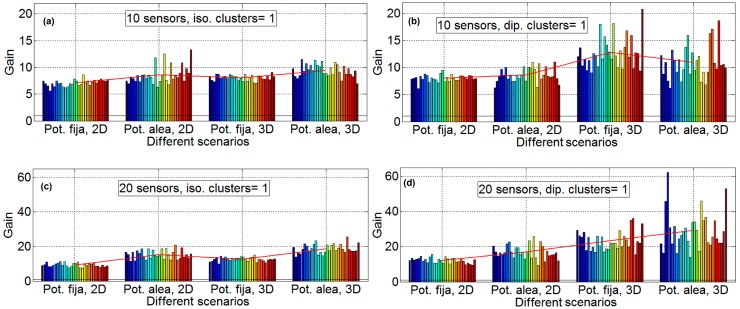
Gain results with one cluster, 10 and 20 sensors, all scenarios. Isotropic antenna and 10 sensors in subfigure (**a**) (**upper left**), dipole antenna and 10 sensors in subfigure (**b**) (**upper right**), isotropic antenna and 20 sensors in subfigure (**c**) (**lower left**), and dipole antenna and 20 sensors in subfigure (**d**) (**lower right**).

Up to now, we have taken into account the gain with beamforming in a certain pointing direction and compared it with the directivity of the sensor in that particular direction. In this section, the received power calculation based on the distance to HECN applying the Friis formula is applied to calculate the propagation loss. The phase center of the array and the exact angle that is the HECN are available, as well as the distance to the central node and the angles of each of the nodes to transmit to the HECN. Simulations have been performed with 10 and 20 sensors, and with one and two clusters. Simulations with 50 sensors have not been done because, as depicted in the previous sections, genetic algorithms with the standard parameters are not able to find a solution when the number of sensors increases. Figure 10 shows the results of performing optimization with one cluster, with 10 and 20 sensors. This figure provides that the performance of the cases with perfect isotropic antenna is very similar to those obtained in the previous section. However, results with an antenna, such as a dipole, are significantly better for 3D cases. This is due to the radiation pattern for the sensors with dipole antennas. As the dipole can have very low directivity in certain directions, if any of these low directivity directions correspond to the exact direction to the HECN station, a low directivity value is obtained. This makes the lifetime of those sensors very low. This problem does not occur when the beamforming technique is applied. Therefore, the gain factor, in some cases, increases up to 45.

**Figure 11:**
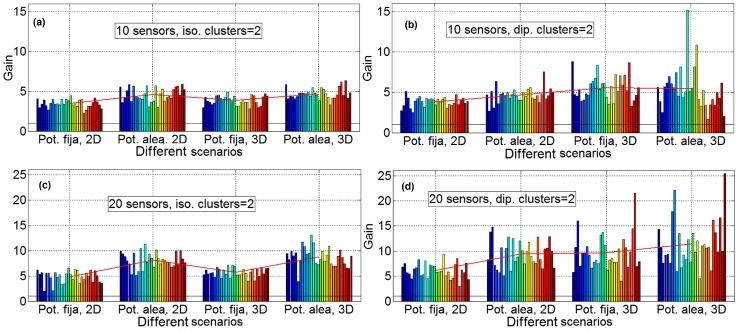
Gain results with two clusters, 10 and 20 sensors, all scenarios. Isotropic antenna and 10 sensors in subfigure (**a**) (**upper left**), dipole antenna and 10 Sensors in subfigure (**b**) (**upper right**), isotropic antenna and 20 sensors in subfigure (**c**) (**lower left**) and dipole antenna and 20 sensors in subfigure (**d**) (**lower right**).

Figure 11 shows the results of performing optimization with two clusters, with 10 and 20 sensors. The results with two clusters for ideal isotropic antenna are very similar to those of the previous section. However, as it occurs in the case of one cluster, the case of two clusters, dipole antenna, and 3D, the gain factor increases in the real case compared to the ideal case. As an example, the mean gain factor obtained in the Scenario D for two clusters is 12 for 20 nodes, while for the same case in Section 4.2 the mean gain factor is equal to 9.

## 6. Conclusions

This work provides the evaluation of WSN node networks and their performance when both clustering and antenna beamforming are applied. In this work we have fixed four different scenarios and each scenario is simulated with different number of sensors implied: 50, 20, 10, five, and two nodes per scenario, where each scenario is randomly generated thirty times in order to validate the results and their repeatability and reliability. For each experiment, two different transmission directions are considered (*φ* = 0° and θ = 45°; *φ* = 45°, and θ = 45°) for the optimization process. Each scenario is evaluated for two different antennas, an ideal isotropic antenna and a conventional dipole one. In this set of experiments, two types of WSN are considered: in the first one, all of the sensors have the same amount of power for communication purposes; in the second one, each sensor has a different amount of available power. The analyzed cases in this document are focused on static nodes (no movement after the random scenario generation) and 2D surface and 3D space for the node location.

In the results, when the desired direction is obtained with beamforming increasing the array directivity, the gain factor for a low number of nodes is equal to the number of sensors, which is an important conclusion of this paper. For a medium number of sensors, the gain factor is equal to the number of sensors only for Scenario D (sensors with different available power for communications and 3D distribution), that is, the scenario with the best performance is the scenario D, which is another important conclusion of this paper. Regarding the number of clusters, it is observed that clusterization is a good strategy for a number of nodes higher than 20. The gain in the two-cluster case is equal to the number of nodes of the cluster. Moreover, in a real environment, with real antennas (dipole), the gain obtained with beamforming is higher for both cases (one and two clusters). To the authors’ knowledge, this is the first time that beamforming and clustering have been simultaneously applied to increase the network performance in WSNs.

Future work is related to an improvement in the optimization algorithms in order to solve adequately scenarios with a high number of sensors. Also, the optimization of the number of clusters and the amplitude and phase of the sensors simultaneously may become another interesting work. Finally, it is possible to develop scenarios with multiple HECN (multiple directions) and multiple directions from which the WSN is being attacked (multiple directions towards which decrease the transmission power).

## Figures and Tables

**Table 1:** Mean gain factor for all scenarios with two antenna and *φ* = 45°, θ = 45°.

(*φ* = 45° θ = 45°)	Scenario A		Scenario B		Scenario C		Scenario D	
N° Sensors	Iso	Dip	Iso	Dip	Iso	Dip	Iso	Dip
2	1.83	1.86	1.80	1.81	1.79	1.81	1.80	1.80
5	4.18	4.19	4.43	4.42	4.20	4.28	4.50	4.52
10	6.51	6.62	9.63	9.75	6.91	7.14	10.16	10.18
20	8.42	8.36	16.41	15.95	10.52	10.57	19.54	19.1
50	8.02	8.06	16.26	15.16	11.34	11.33	21.66	21.93
